# Low socioeconomic status and severe obesity are linked to poor cognitive performance in Malaysian children

**DOI:** 10.1186/s12889-019-6856-4

**Published:** 2019-06-13

**Authors:** Bee Koon Poh, Shoo Thien Lee, Giin Shang Yeo, Kean Choon Tang, Ab. Rahim Noor Afifah, Awal Siti Hanisa, Panam Parikh, Jyh Eiin Wong, Alvin Lai Oon Ng, on behalf of the SEANUTS Study Group, Bee Koon Poh, A. Karim Norimah, Abd Talib Ruzita, Siti Balkis Budin, Alvin Lai Oon Ng, Mohd Din Siti Haslinda, Jyh Eiin Wong, Mohd Noor Ismail, Jamal Rahman, Nor Azmi Kamaruddin, Safii Nik Shanita, Yit Siew Chin, Bee Suan Wee, Nor Aini Jamil

**Affiliations:** 1 Nutritional Sciences Programme & Centre for Community Health, Faculty of Health Sciences, Universiti Kebangsaan Malaysia, Jalan Raja Muda Abdul Aziz, 50300 Kuala Lumpur, Malaysia; 2 FrieslandCampina, Amersfoort, LE 3818 The Netherlands; 3 Department of Psychology, School of Science and Technology, Sunway University, 47500 Petaling Jaya, Malaysia

**Keywords:** Child, Cognition, Economic status, Intelligence, Obesity, Malaysia, Nutritional status

## Abstract

**Background:**

Socioeconomic factors and nutritional status have been associated with childhood cognitive development. However, previous Malaysian studies had been conducted with small populations and had inconsistent results. Thus, this present study aims to determine the association between socioeconomic and nutritional status with cognitive performance in a nationally representative sample of Malaysian children.

**Methods:**

A total of 2406 Malaysian children aged 5 to 12 years, who had participated in the South East Asian Nutrition Surveys (SEANUTS), were included in this study. Cognitive performance [non-verbal intelligence quotient (IQ)] was measured using Raven’s Progressive Matrices, while socioeconomic characteristics were determined using parent-report questionnaires. Body mass index (BMI) was calculated using measured weight and height, while BMI-for-age Z-score (BAZ) and height-for-age Z-score (HAZ) were determined using WHO 2007 growth reference.

**Results:**

Overall, about a third (35.0%) of the children had above average non-verbal IQ (high average: 110–119; superior: ≥120 and above), while only 12.2% were categorized as having low/borderline IQ (< 80). Children with severe obesity (BAZ > 3SD), children from very low household income families and children whose parents had only up to primary level education had the highest prevalence of low/borderline non-verbal IQ, compared to their non-obese and higher socioeconomic counterparts. Parental lack of education was associated with low/borderline/below average IQ [paternal, OR = 2.38 (95%CI 1.22, 4.62); maternal, OR = 2.64 (95%CI 1.32, 5.30)]. Children from the lowest income group were twice as likely to have low/borderline/below average IQ [OR = 2.01 (95%CI 1.16, 3.49)]. Children with severe obesity were twice as likely to have poor non-verbal IQ than children with normal BMI [OR = 2.28 (95%CI 1.23, 4.24)].

**Conclusions:**

Children from disadvantaged backgrounds (that is those from very low income families and those whose parents had primary education or lower) and children with severe obesity are more likely to have poor non-verbal IQ. Further studies to investigate the social and environmental factors linked to cognitive performance will provide deeper insights into the measures that can be taken to improve the cognitive performance of Malaysian children.

## Background

Early childhood development has far reaching consequences on an individual’s cognitive performance, which in turn affects his or her lifelong productivity, socioeconomic status (SES) and health. Cognitive performance involves the adaptive mental processes of perception, reasoning, creativity, problem solving and intuition that are measured by intelligence quotient (IQ) [1]. Poor cognitive development and low IQ levels among children may eventually lead to problems in mental health [2], social development, peer relationships as well as physical health [3], all of which can subsequently affect their quality of life when they are adults [4].

Poor cognitive performance in children has been linked with multiple risk factors related to low SES, such as parental education level and in particular low maternal schooling [5, 6], malnutrition, micronutrient deficiencies [7], non-stimulating environment [8], childhood infections [9, 10] and hearing impairment [11].

SES is a multidimensional construct typically characterized by education, income and occupation [12]. Results from developed and developing countries consistently supported the links among SES, nutritional status and cognitive performance [13–23]. Nutritional status, an associated factor of SES [13, 14], also plays a crucial rule in predicting cognitive performance. Good nutrition provides the building blocks for brain and neural system development [15]. Studies have regularly linked cognitive performance with both over- and under-nutrition. Sandjaja et al. [16] reported that both under- and over-nutrition can contribute to poor cognitive performance among Southeast Asian children aged 7–12 years. Another study has associated increased body mass index (BMI) with poor cognitive performance among children and adolescents aged 8 to 16 years in the United States [17]. An Indonesian study found that height-for-age Z-score (HAZ) of preschool children was positively associated with cognitive development [18]; while another study showed that stunted Indonesian children had slower development in fine and gross motor skills as well as poorer language skills, compared to non-stunted children [19]. Undernutrition in children is known to have long-term adverse effects on cognitive performance, school completion and productivity during adulthood [20]. Studies from developing countries, such as Vietnam [21], Indonesia [22] and Guatemala [23], have reported similar results.

Studies conducted in Malaysia support the relationship between SES, nutritional status and cognitive performance. Parental education, household income, birth weight, micronutrient deficiencies and intestinal parasitic infections have been identified as major risk factors for cognitive performance in Malaysian preschool [24] and primary schoolchildren [1, 7]**.** However, such studies were limited to indigenous children or primary school-aged children from particular locations in the country. This present study, therefore, aims to determine the association between SES and nutritional status with cognitive performance in a nationally-representative sample of Malaysian children.

## Methods

### Study design

The present study uses Malaysian data from the South East Asian Nutrition Surveys (SEANUTS), which was conducted among children in four countries, namely Indonesia, Malaysia, Thailand and Vietnam. SEANUTS Malaysia was a nationally-representative cross-sectional study conducted among children aged 6 months to 12 years using stratified sampling in all six regions of Malaysia, namely the Northern, Central, Southern, and East Coast regions of Peninsular Malaysia, as well as Sabah and Sarawak [25]. This analysis included a total of 2406 children aged 5 to 12 years (who had complete data for cognitive assessment), representing an estimated 3.55 million Malaysian children in the same age range. Among these children, 631 (24.3%) were preschool children aged 5 and 6 years old, while 1775 were primary schoolchildren aged 7 to 12 years old.

This study was conducted according to the guidelines laid down in the Declaration of Helsinki, and all procedures involving human subjects were approved by the Research Ethics Committee of Universiti Kebangsaan Malaysia (Project Code: NN-072-2009). Permission to conduct the study was obtained from the Ministry of Education Malaysia and the relevant State Education Departments. Written informed consent was obtained from the parents or guardians of all participants prior to data collection. Details of the study design and sampling protocol have been described elsewhere [25, 26]. This project was registered in the Dutch Trial Registry as NTR2462.

### Socioeconomic status

Socioeconomic information, such as age, sex, ethnicity, parents’ education and monthly household income, was obtained from the parents or guardians using a self-administered questionnaire. Parental education was categorized into: (i) non-schooling and primary school, (ii) secondary school and (iii) tertiary level. Household income in Malaysian Ringgit (MYR) was categorized into four groups, using criteria set forth in the Tenth Malaysia Plan [27]: (i) very low income: below MYR 1500 per month; (ii) low income: between MYR 1500 and MYR 2299 per month; (iii) middle income: between MYR 2300 and MYR 5599 per month and (iv) high income: MYR 5600 or more per month [USD 1 = MYR 4.1405 (as at 28 September 2018)].

### Cognitive performance

Trained research assistants administered age-appropriate, validated psychometric Raven’s Progressive Matrices (RPM) to assess the non-verbal intelligence quotient (IQ) of the children. Care was taken to administer the RPM to the children individually in a comfortable room that was well lit and free from noise. For children aged 5 to 11 years, Coloured Progressive Matrices (CPM; Raven) [28] were used and Standard Progressive Matrices (SPM; Raven) [29] were administered to children aged 12 years. The CPM consist of three sets of 12 problems, while the SPM consist of five sets of problems, with each set becoming progressively more difficult. Each correct answer was given a score of 1, making a total raw score of 36 for CPM and 60 for SPM. The total raw scores were then converted into a standard score based on norm tables, and subsequently categorized into the relevant non-verbal IQ categories: ≥120 (superior); 110–119 (high average); 90–109 (average), 80–89 (below average); < 80 (low/borderline) [28, 29].

### Anthropometric status

Anthropometric measurements, including body weight and height were measured by trained research assistants. The measurements were taken with the children wearing light clothing and not wearing shoes. Height was measured to the nearest 0.1 cm, with a portable SECA stadiometer Model 213 (SECA, Hamburg, Germany). Body weight was taken to the nearest 0.1 kg using a SECA digital weighing scale Model 803 (SECA, Hamburg, Germany). Measurements were taken twice and the mean was calculated. Body mass index (BMI) was calculated by dividing the measured weight (kg) by the square of height (m^2^).

Anthropometric status was classified according to the age- and sex-specific WHO [30] growth reference using the WHO AnthroPlus 1.0.3 (World Health Organisation, Geneva, Switzerland). The cut off values for thinness was BAZ < -2SD, while overweight and obesity were > 1SD and > 2SD, respectively. Severe obesity was defined as BAZ > 3SD. The cut off value for stunting was HAZ < -2SD [30].

### Data analysis

Data was analyzed using complex samples technique in SPSS version 20.0 (IBM Corporation, New York, USA), using a sampling weight factor developed based on the Malaysian population census 2010 [31]. Descriptive statistics, including mean, standard error (SE), percentage and 95% confidence interval (CI), were used to describe sociodemographic characteristics, nutritional status and cognitive levels. Likelihood-ratio tests were used to test the association of socioeconomic and nutritional status with IQ categories. The difference in IQ distribution of children by SES and nutritional status was described by percentages and 95% confidence interval (CI) estimates.

Independent variables which produce likelihood ratio with *p*-value of 0.2 and below in univariate analyses, or change the odds ratio of the variable of interest by 10% or more, were included in the multivariate logistic regression model. Complex samples logistic regression analyses were performed to determine the odds ratio (OR) after adjusting for putative confounding variables. The OR represents the probability of getting lower IQ relative to those with high average/superior IQ (reference group).

Two regression models were presented. Model 1 was unadjusted with household income, paternal and maternal education, BAZ categories as the primary independent variables. Model 2 was further adjusted by sex, age, ethnicity and residence as these factors had been previously reported to influence children’s cognition [7, 32]. The logistic regression models were also checked for the moderating effect of sex and age group on association with IQ levels. Due to insignificant interaction terms (*p* > 0.05), the regression models were presented without stratification. The significance level was set as *p* < 0.05 using two-sided tests for all analysis.

## Results

Table 1 illustrates the sample characteristics according to SES, anthropometric status and non-verbal IQ. Mean age of the children was 9.0 ± 0.1 years. Nearly 59.1% were Malays, followed by Chinese (19.2%), Other ethnicities (15.0%) and Indians (6.7%). A third of the children were from very low income households (< MYR1500 per month) and less than one fifth belonged to high income households (≥ MYR5600 per month). About two-thirds of the children had parents who had completed secondary school education (fathers: 64.6%; mothers: 66.4%). The proportion of children who were stunted, thin and severely obese were 6.0%, 6.9% and 4.9%, respectively.

**Table 1:** Sociodemographic characteristics, nutritional status and intelligence quotient (IQ) of children aged 5.0 to 12.9 years

	Unweighted count (n)	Estimated population	Percentage (%)	95% CI	Mean	SE
All	2406	3,548,653				
Age (years)					9.0	0.1
Sex						
Boys	1201	1,823,998	51.4	48.7, 54.0		
Girls	1205	1,724,655	48.6	46.0, 51.3		
Age groups						
5.0–6.9 years	631	865,374	24.3	22.3, 26.6		
7.0–9.9 years	892	1,339,714	37.8	35.3, 40.3		
10.0–12.9 years	883	1,343,566	37.9	35.2, 40.6		
Residence						
Urban	1440	2,833,601	79.9	78.0, 81.5		
Rural	966	715,052	20.1	18.5, 22.0		
Income groups						
Below MYR1500	863	1,104,249	31.1	28.7, 33.7		
MYR1500-MYR2299	434	707,382	19.9	17.8, 22.2		
MYR2300-MYR5599	712	1,111,841	31.3	29.0, 33.8		
MYR5600 and above	397	625,182	17.7	15.7, 19.7		
Paternal education level						
Non-schooling and primary school	221	281,450	7.9	6.5, 9.7		
Secondary school	1577	2,290,524	64.6	62.0, 67.1		
Tertiary school	608	976,680	27.5	25.2, 29.9		
Maternal education level						
Non-schooling and primary school	193	248,762	7.0	5.7, 8.7		
Secondary school	1604	2,354,731	66.4	63.8, 68.8		
Tertiary school	609	945,161	26.6	24.4, 29.0		
Ethnicity						
Malay	1148	2,098,763	59.1	56.6, 61.6		
Chinese	593	682,464	19.2	17.5, 21.1		
Indian	172	236,120	6.7	5.5, 8.0		
Others	493	531,307	15.0	13.4, 16.6		
Anthropometry						
Weight (kg)					31.0	0.4
Height (cm)					130.2	0.4
Body mass index (BMI)(kg/m^2^)					17.6	0.1
BMI-for-age Z-score (BAZ)					0.2	0.1
Height-for-age Z-score (HAZ)					−0.4	0.1
Nutritional status						
Thinness	161	244,094	6.9	5.6, 8.5		
Normal weight	1548	2,245,077	63.3	60.6, 65.8		
Overweight	314	462,766	13.0	11.3, 15.0		
Obese	270	423,983	11.9	10.2, 14.0		
Severe obesity	113	172,734	4.9	3.9, 6.0		
Stunted	158	213,483	6.0	4.9, 7.4		
Intelligence quotient (IQ)						
Low/borderline	324	433,580	12.2	10.6, 14.0		
Below average	307	485,845	13.7	11.8, 15.8		
Average	913	1,388,445	39.1	36.6, 41.8		
High average	451	651,509	18.4	16.5, 20.4		
Superior	411	589,275	16.6	14.8, 18.6		

IQ categories: < 80, low/borderline; 80–90, below average; 90–109, average; 110–119, high average; ≥ 120, superior

Other ethnic groups include Sarawak bumiputra, Sabah bumiputra, and other bumiputra

ǂUSD 1 = MYR 4.1405 (as at 28 September 2018)

Four out of ten children (39.1%) had average non-verbal IQ. A third of the children (35.0%) had above average (high average and superior) non-verbal IQ, while an eighth (12.2%) were categorized as having low or borderline IQ (Table 1). The distribution of the children’s non-verbal IQ categories by sociodemographic characteristics and nutritional status is shown in Table 2. A larger proportion of children from families with very low household income had low/borderline IQ (17.3%), while high income households had a larger proportion of children with superior IQ (29.4%). The same is true for parental education level, where a higher proportion of children whose parents had the lowest education level were categorized as having low/borderline non-verbal IQ (paternal: 17.7%; maternal: 21.8%), and, in contrast, a higher proportion of children whose parents had tertiary education were categorized as having superior non-verbal IQ (paternal: 25.4%; maternal: 26.2%). In terms of ethnic groups, Chinese children had the lowest proportion of low/borderline non-verbal IQ (7.5%) and the highest proportion of superior non-verbal IQ (28.5%). There was no significant association of BAZ and HAZ with IQ categories.

**Table 2:** Distribution (%) of children’s intelligence quotient (IQ) by sociodemographic characteristics and nutritional status categories

	Low/borderline		Below average		Average		High average		Superior		Likelihood ratio	*p* value
	Mean	95% CI	Mean	95% CI	Mean	95% CI	Mean	95% CI	Mean	95% CI		
Sex												
Boys	10.1	8.3, 12.2	14.3	11.6, 17.5	38.0	34.5, 41.7	20.7	17.9, 23.7	16.9	14.5, 19.7	18.726	p < 0.05
Girls	14.5	11.9, 17.6	13.1	10.6, 16.0	40.3	36.6, 44.1	15.9	13.5, 18.6	16.3	13.6, 19.3		
Age groups												
5.0–6.9 years	14.6	11.3, 18.7	11.9	9.2, 15.4	36.0	31.5, 40.7	19.4	15.9, 23.4	18.1	14.8, 22.0	37.301	*p* < 0.01
7.0–9.9 years	12.4	10.1, 15.1	13.5	11.0, 16.5	36.2	32.3, 40.3	17.6	14.8, 20.9	20.2	17.1, 23.8		
10.0–12.9 years	10.5	7.9, 13.8	15.0	11.4, 19.4	44.1	39.4, 48.9	18.4	15.3, 22.0	12.0	9.4, 15.2		
Residence												
Urban	11.9	10.0, 14.1	13.1	10.9, 15.6	38.8	35.8, 42.0	17.9	15.8, 20.3	18.2	16.0, 20.6	21.290	p < 0.01
Rural	13.3	11.0, 16.1	16.2	12.9, 20.1	40.3	36.0, 44.7	20.0	16.5, 24.1	10.2	8.2, 12.7		
Income groups												
Below MYR1500	17.3	14.0, 21.1	17.5	13.6, 22.2	43.2	38.4, 48.1	13.9	11.2, 17.1	8.2	6.2, 10.7	151.781	*p* < 0.001
MYR1500-MYR2299	12.9	9.3, 17.7	13.9	10.3, 18.5	37.9	32.0, 44.2	21.5	17.0, 26.9	13.8	10.4, 18.1		
MYR2300-MYR5599	9.9	7.5, 13.0	10.9	8.4, 14.1	40.9	36.5, 45.5	18.7	15.6, 22.3	19.6	16.3, 23.3		
MYR5600 and above	6.5	4.2, 10.1	11.8	7.9, 17.3	30.2	25.0, 35.9	22.1	17.3, 27.6	29.4	23.9, 35.7		
Paternal education level												
Non-schooling and primary school	17.7	11.9, 25.6	22.4	13.3, 35.3	36.4	27.2, 46.7	14.4	9.2, 21.7	9.1	5.4, 15.0	99.370	p < 0.001
Secondary school	14.2	12.0, 16.7	13.6	11.5, 16.0	41.0	37.7, 44.3	17.4	15.2, 19.9	13.8	11.8, 16.0		
Tertiary school	6.0	4.0, 8.7	11.3	8.2, 15.4	35.6	31.1, 40.4	21.7	18.0, 25.9	25.4	21.2, 30.1		
Maternal education level												
Non-schooling and primary school	21.8	14.4, 31.6	21.0	12.3, 33.4	37.9	27.7, 49.1	10.1	6.0, 16.5	9.3	4.9. 17.1	119.384	p < 0.001
Secondary school	13.5	11.5, 15.9	13.7	11.5, 16.2	42.2	39.0, 45.5	17.1	14.9, 19.5	13.5	11.6, 15.7		
Tertiary school	6.4	4.5, 9.0	11.9	8.7, 15.9	31.7	27.3, 36.5	23.8	19.9, 28.2	26.2	22.0, 30.9		
Ethnicity												
Malay	13.5	11.2, 16.2	16.1	13.3, 19.3	41.7	38.0, 45.5	15.5	13.0, 18.3	13.2	10.9, 16.0	117.651	p < 0.001
Chinese	7.5	5.4, 10.2	8.2	6.0, 11.1	30.5	26.3, 35.1	25.3	21.3, 29.8	28.5	24.2, 33.2		
Indian	17.6	12.1, 24.9	12.0	7.4, 19.0	45.2	35.5, 55.3	13.0	8.3, 19.7	12.1	6.8, 20.8		
Others	10.9	7.3, 16.0	12.0	8.5, 16.6	37.3	32.2, 42.7	23.2	19.2, 27.7	16.6	13.1, 20.8		
BAZ groups												
Thinness	5.9	3.3, 10.3	18.1	11.7, 26.9	44.8	34.1, 56.1	16.6	11.0, 24.4	14.6	8.9, 22.8	43.447	*p* = 0.102
Normal weight	12.5	10.6, 14.7	11.5	9.6, 13.8	39.3	36.2, 42.6	18.7	16.4, 21.3	17.9	15.7, 20.4		
Overweight	14.9	10.6, 20.7	14.7	9.1, 23.0	38.2	31.3, 45.5	19.3	14.5, 25.2	12.9	8.9, 18.4		
Obese	9.4	4.7, 17.6	19.4	13.0, 28.0	37.5	30.1, 45.5	19.9	14.5, 26.6	13.9	8.5, 21.8		
Severe obesity	17.1	9.9, 28.0	19.0	11.4, 29.9	35.1	25.4, 46.2	10.1	5.6, 17.5	18.7	11.9, 28.3		
HAZ groups												
Stunted	16.4	9.9, 26.2	14.8	9.0, 23.4	36.6	26.4, 48.2	19.4	12.3, 29.1	12.8	7.7, 20.4	3.969	*p* = 0.656
Normal height	11.9	10.3, 13.8	13.6	11.7, 15.8	39.3	36.6, 42.0	18.3	16.4, 20.4	16.8	15.0, 18.9		

IQ categories: < 80, low/borderline; 80–90, below average; 90–109, average; 110–119, high average; ≥ 120, superior

Other ethnic groups include Sarawak bumiputra, Sabah bumiputra, and other bumiputra

ǂUSD 1 = MYR 4.1405 (as at 28 September 2018)

Table 3 shows that the OR of logistic regression models improved after adjusting for covariates. Children from households with very low income had twice the odds of having poor non-verbal IQ [low/borderline/below average, OR = 2.01, (95%CI 1.16, 3.49)], when compared with children from high-income families. The odds of having poor IQ level also doubled among children whose parents did not attend school or who had completed only primary education, compared with children whose parents had completed tertiary education [paternal, OR = 2.38 (95%CI 1.22, 4.62); maternal, OR = 2.64 (95%CI 1.32, 5.30)]. Besides, children whose fathers had completed secondary education had 63% higher risk of having poor non-verbal IQ level [low/borderline/below average, OR = 1.63 (95%CI 1.06, 2.52)]. The odds of children with severe obesity having poor non-verbal IQ were twice as high compared with normal weight children [OR = 2.28 (95%CI 1.23, 4.24)].

**Table 3:** Odds ratio for intelligence quotient (IQ) by sociodemographic characteristics and nutritional status

		Unadjusted model^a^		Adjusted model^a^	
		OR	95% CI	OR	95% CI
Income groups^b^					
Below MYR1500	Low/borderline/below average	2.51^*^	1.47, 4.27	2.01^*^	1.16, 3.49
	Average	2.27^*^	1.45, 3.54	1.95^*^	1.24, 3.06
	High average/superior	1		1	
MYR1500-MYR2299	Low/borderline/below average	1.34	0.78, 2.30	1.15	0.66, 2.01
	Average	1.30	0.82, 2.07	1.18	0.74, 1.90
	High average/superior	1		1	
MYR2300-MYR5599	Low/borderline/below average	1.14	0.71, 1.84	1.06	0.65, 1.72
	Average	1.44	0.99, 2.09	1.38	0.95, 2.02
	High average/superior	1		1	
Paternal education level^c^					
Non-schooling and primary school	Low/borderline/below average	1.78	0.93, 3.43	2.38^*^	1.22, 4.62
	Average	0.90	0.51, 1.60	1.11	0.63, 1.97
	High average/superior	1		1	
Secondary school	Low/borderline/below average	1.42	0.93, 2.17	1.63^*^	1.06, 2.52
	Average	1.01	0.73, 1.40	1.12	0.80, 1.55
	High average/superior	1		1	
Maternal education level^c^					
Non-schooling and primary school	Low/borderline/below average	2.50^*^	1.25, 5.00	2.64^*^	1.32, 5.30
	Average	2.07^*^	1.07, 4.02	2.12^*^	1.10, 4.08
	High average/superior	1		1	
Secondary school	Low/borderline/below average	1.41	0.95, 2.10	1.34	0.89, 2.02
	Average	1.63^*^	1.17, 2.28	1.55^*^	1.11, 2.17
	High average/superior	1		1	
BAZ groups^d^					
Thinness	Low/borderline/below average	0.94	0.54, 1.64	0.79	0.45, 1.41
	Average	1.23	0.74, 2.03	1.05	0.64, 1.73
	High average/superior	1		1	
Overweight	Low/borderline/below average	1.44	0.92, 2.26	1.45	0.93, 2.27
	Average	1.12	0.78, 1.62	1.08	0.74, 1.57
	High average/superior	1		1	
Obese	Low/borderline/below average	1.32	0.83, 2.10	1.33	0.82, 2.17
	Average	1.09	0.74, 1.60	1.06	0.69, 1.61
	High average/superior	1		1	
Severe obesity	Low/borderline/below average	2.08^*^	1.14, 3.77	2.28^*^	1.23, 4.24
	Average	1.21	0.70, 2.11	1.28	0.73, 2.24
	High average/superior	1		1	

^*^Significant odds ratio using complex sample logistic regression at *p* < 0.05

^a^Reference category of IQ groups is high average/superior. Sex, ethnicity, age and residence areas (rural/urban) were adjusted in the adjusted model

^b^Reference category of income groups is high income group (MYR5600 and above). ǂUSD 1 = MYR 4.1405 (as at 28 September 2018)

^c^Reference category of paternal and maternal education level is tertiary education level

^d^Reference category of BAZ groups is normal weight

## Discussion

Our results confirm the association between low SES, in particular low household income and parental education, and poorer cognitive functioning in Malaysian children aged 5–12 years. Children with severe obesity had twice the odds of having poorer non-verbal IQ performance compared to their normal weight counterparts. However, being stunted was not associated with cognitive performance of the children.

In line with previous studies, our study shows that children from low household income families [33, 34] and whose parents had low education levels [33, 35] tended to score lower on the Raven’s non-verbal IQ test. Household financial constraints had been associated with such conditions as limited access to cognitively stimulating materials and limited preschool experiences for children [36, 37]. Children from low SES families are more likely to have poorer cognitive performance possibly because of their parents’ behavior and life decisions. As people may behave differently when they perceive that required resources are scarce [38], it has been suggested that there is a higher likelihood of poor people engaging in less healthy activities, including tobacco use [39] and alcohol consumption [40]. Hence, different priorities when budgeting limited income may have led to parents with less income not providing the necessary stimulation needed for nurturing their children’s cognitive functioning.

On the contrary, parents with higher education may be more willing to invest time and money in caring for their children [41]. In addition, parents with higher education usually have higher health literacy and engage in quality interactions more frequently with their children, as compared to parents with lower education. This explanation is in agreement with that from a Spanish study, which suggested that active parental involvement in the parent-child relationship and the parents’ willingness to interact with their children are highly dependent on household SES [42]. A major U.S study showed that parental education was linearly associated with children’s total brain surface area [43], which is an indicator for intelligence [44]. Apart from psychosocial factors within the family setting, genetic heritage may also be an important contributing factor towards children’s cognitive abilities.

The main effect of severe obesity on cognitive performance seems to corroborate the earlier findings of Sandjaja et al. [16], which reported a similar relationship among Southeast Asian children. However, the negative effect of being overweight or obese on cognitive development as reported by Sandjaja et al. [16] was not shown in this study. There are several possible explanations for the link between severe obesity and non-verbal IQ. One possible explanation is related to nutrition, for example, adequate intake of macro- and micro-nutrients, which is key to brain and cognitive growth, particularly during early childhood. Obesity may indicate adequacy of energy-dense foods but not necessarily the sufficiency of such micronutrients as iron, iodine, zinc and vitamin B12 which have crucial roles in neuropsychological development for cognitive performance [45]. Therefore, it is important that children consume adequate but not excessive macronutrients and sufficient micronutrients, as these nutrients are essential for cognitive development [15]. Furthermore, children with severe obesity may intensify the adverse effect of adiposity [46]. Higher adipose tissues can result in higher adipokines production, including leptin [47]. Adipokine increases insulin resistance and therefore promotes hyperinsulinemia, dyslipidemia, inflammation and endothelial dysfunction [48]. Hypertriglyceridemia (one of the dyslipidemias) will result in elevated peripheral leptin levels, which prevent the entry of leptin to the brain, thus harming brain development [48, 49], and consequently lowers cognitive performance.

The finding that severe obesity is associated with low cognitive performance can also be explained by the tendency of severely obese children to have low physical activity levels [50], possibly due to more physical and social barriers to engage in physical activity, compared to their normal weight peers [51]. The lack of physical activity has been associated with poorer cognitive performance, including executive control, working memory and cognitive flexibility in children [52]. Lack of social environment support may also discourage participation in physical activity among children who are obese [53], thus leading to poorer cognitive development.

Notably, our study does not find any association between stunting and cognitive performance. This is in contrast to previous studies which had found that stunted children had lower cognitive performance [18, 54]. However, it is possible that the potential adverse effects of stunting may have been attenuated by high quality home learning environments [6, 21]. An early childhood study in Vietnam found that the cognitive development disadvantages associated with stunting were found among children whose home learning environments were of low quality, but was absent among children that have good home learning environment [21]. This is supported by a Jamaican study, which reported that stunted children who received home stimulation treatment had significantly superior and longer lasting benefits on cognition compared with those who were only provided with nutrition supplements [6]. Hence, our finding implies that in order to tackle the issues related to cognitive development of children in Malaysia, more attention should be focussed on towards improving not only nutrition but also the factors related to developing stimulating home and learning environment.

The strength of the present study is that by employing a nationally representative sample of Malaysian children in this analysis, we are able to provide, for the first time, insights into the cognitive performance of Malaysian children aged 5 to 12 years and its related factors. However, the generalisability of these results is subject to several limitations. First, due to the cross-sectional design of the study, it is not possible to infer causal relationship between SES or nutritional status with cognitive function. Second, the assessment of cognitive performance was restricted to only non-verbal IQ using RPM, which measures the logical reasoning part of intelligence. Future studies should also include assessment of other components of intelligence that are more representative of basic adaptive skills in social settings, such as verbal comprehension and social reasoning. Besides, this study focuses only on SES and nutritional status as determinants of cognitive function. Further investigations into contextual variables may be required to account for other psychosocial and environmental factors – access to cognitively stimulating materials, types of preschool experiences and parent-child interactions [36, 37, 42] – that affect the cognitive performance of children. Examining the cognitive functioning and behavioral patterns of children from diverse demographic groups may offer further insights into understanding the interplay between the sociodemographic, psychosocial and environmental factors that influence the cognitive performance of children.

## Conclusions

Household income, parental education level and nutritional status are associated with the cognitive performance of 5-to-12 year-old Malaysian children. This study highlights that children from lower socioeconomic classes and those with severe obesity are disadvantaged and are more likely to have poor cognitive performance. The findings of this study indicate the need for further investigation of the interrelated influences between SES and health behaviours, as well as the social and environmental factors that can improve the nutritional status and cognitive health of Malaysian children.

## Abbreviations

BAZ: BMI-for-age Z-score
BMI: Body Mass Index
CI: Confidence Interval
CPM: Coloured Progressive Matrices
HAZ: Height-for-age Z-score
IQ: Intelligence Quotient
OR: Odds Ratio
RPM: Raven’s Progressive Matrices
SE: Standard Error
SEANUTS: South East Asian Nutrition Surveys
SES: Socioeconomic Status
SPM: Standard Progressive Matrices
